# Association of chorioamnionitis with infertility treatment and subsequent neonatal outcomes in the US: a population-based cohort study

**DOI:** 10.1186/s12884-023-05619-0

**Published:** 2023-05-20

**Authors:** Meng Ni, Lijuan Li, Qianqian Zhang, Jiuru Zhao, Wei Li, Qianwen Shen, Dongting Yao, Tao Wang, Baihe Li, Xiya Ding, Sudong Qi, Zhiwei Liu

**Affiliations:** 1 International Peace Maternity and Child Health Hospital, School of Medicine, Shanghai Jiao Tong University, Shanghai, China; 2 International Peace Maternity and Child Health Hospital of China Welfare Institution, Shanghai Jiao Tong University, Shanghai, China; 3 Shanghai Key Laboratory of Embryo Original Disease, Shanghai, China

**Keywords:** Infertility treatment, Chorioamnionitis, Neonatal outcomes, Preterm birth, Low birth weight

## Abstract

**Background:**

Chorioamnionitis (CAM) is a common risk factor for preterm births, resulting in several adverse outcomes. The association between infertility treatment and CAM is unclear. Therefore, this study examined the association between infertility treatment and CAM and described subsequent neonatal outcomes.

**Methods:**

This population-based cohort study used data from the National Vital Statistics System Database. We included women who had a singleton live birth from January 1, 2016 to December 31, 2018. Women-infant pairs were stratified by infertility treatment, and the main outcome was a reported diagnosis of CAM in a checkbox format: clinical CAM or maternal temperature of > 38 °C. Multivariate logistic regression was used to examine the association between infertility treatment and CAM and the effect of infertility treatment on neonatal outcomes in women diagnosed with CAM.

**Results:**

The final sample comprised 10,900,495 woman-infant pairs, and 1.4% received infertility treatment. Compared with the natural conception group, women receiving infertility treatment had a significantly higher risk of CAM (adjusted odds ratio [aOR] 1.772 [95% confidence interval {CI}, 1.718–1.827]). Furthermore, newborns exposed to CAM had a higher risk of very low birth weight (VLBW) (aOR, 2.083 [95% CI, 1.664–2.606], *P* < .001), preterm birth (aOR, 1.497 [95% CI, 1.324–1.693]; *P* < .001), neonatal intensive care unit admission (aOR, 1.234 [95% CI, 1.156–1.317]; *P* < .001), and other adverse neonatal outcomes in the infertility treatment group compared with ones conceived naturally.

**Conclusions:**

This study found that women who received infertility treatment had a higher risk of CAM. And CAM deteriorated neonatal outcomes in the infertility treatment group.

**Supplementary Information:**

The online version contains supplementary material available at 10.1186/s12884-023-05619-0.

## Introduction

As of 2020, 10.09% of all births in the United States were premature [1], resulting in 75% perinatal mortality and half long-term morbidity [2]. In addition, 40–70% of premature births are induced by chorioamnionitis (CAM), particularly in early gestation [3, 4]. Acute CAM or intra-uterine inflammation indicates that a pregnant woman is exposed to inflammatory or infectious disorders of the chorion or amnion. Either of these conditions could lead to an increased risk of developing serious complications [5] such as sepsis [6], cerebral palsy [7], and bronchopulmonary dysplasia in the mother-infant pair [8].

Infertility is defined as 1 year of unwanted non-conception with unprotected intercourse in the fertile phase of menstrual cycles [9] and affects 8–12% of the population worldwide [10]. Consequently, assisted reproductive technology (ART) has become widespread since the early 1980s; this technology includes in vitro fertilization (IVF) and intracytoplasmic sperm injection (ICSI) [11]. Non-ART treatments, consisting of fertility medications, artificial insemination, and intrauterine insemination, result in 4.6% of United States (US) births, which is four times greater than the contribution of ART [12]. Despite great progress, newborns after IVF/ICSI have more compromised perinatal outcomes than spontaneously conceived newborns [13], such as preterm birth [14] and defects in neurodevelopmental health [15] and cardiovascular function and metabolism [16].

Moreover, ART is associated with an abnormal condition in the mother-infant surface modulated by the placenta, which might contribute to a hyper/hyporeactive status to infection or other inflammations [17]. CAM has been demonstrated to have an infection/inflammation status before delivery; however, the association between infertility treatment and CAM has not been clarified. One study found that women with mixed infertility who conceived by IVF had a higher risk of CAM among singleton pregnancies than those who conceived spontaneously [18]. However, few studies have considered the maternal infection status during pregnancy when evaluating the relationship between infertility treatment and CAM. Additionally, further studies are necessary to determine if infertility treatment deteriorates CAM-related neonatal outcomes.

In this study, we explored US birth certificate data to examine the association between infertility treatment and CAM and the subsequent neonatal outcomes in the population with CAM. This can provide information to obstetricians and neonatologists when evaluating women and infants receiving infertility treatment.

## Methods

### Study population

In this prospective, population-based cohort study, we explored the National Vital Statistics System database and collected birth and death records submitted by 50 states and the District of Columbia to the Centers for Disease Control (CDC). This study included 1,162,440 mothers in the database with live births from January 1, 2016 to December 31, 2018. After excluding 10,310 missing obstetric complications records, 396,031 with twins or multiple births, 208,169 with pre-pregnancy hypertension, 84,622 with diabetes, and 33,056 with incomplete medical records, a total of 10,900,495 mother-infant pairs were finally recruited. According to the International Peace Maternal and Child Hospital Institutional Review Board, this study was exempt from the requirement for informed consent because the data used were publicly available. We followed the Strengthening the Reporting of Observational Studies in Epidemiology guidelines.

### Exposure and outcomes

The main outcome was a diagnosis of CAM during the present pregnancy after infertility treatment, which was identified in a checkbox format: clinical CAM or maternal temperature > 38 °C.

We included several neonatal outcomes including sex, gestational age, birth weight, neonatal intensive care unit (NICU) admission, assisted ventilation, assisted ventilation > 6 h, and surfactant or antibiotic use. Neonatal sex was categorized as male or female. Gestational age was calculated using obstetric estimation at delivery as preterm (< 37 weeks) and a specified category: extremely (delivery < 28 weeks), very (delivery at 28–31^+6^ weeks), and moderate and late (delivery at 32–36^+6^ weeks). Birth weight was classified as normal, low (LBW, 1500 g -2500 g), and very low (VLBW, ≤ 1500 g).

The exposure in this study was infertility treatment, including (1) ART, such as IVF, intrafallopian gamete transfer, and zygote intrafallopian transfer, and (2) non-ART treatment, such as fertility-enhancing drugs, artificial insemination, and intrauterine insemination. Mothers who received both ART and non-ART treatment were classified into the ART group. In the main analysis, both (1) and (2) were considered infertility treatment groups. For the subgroup analysis, (1) and (2) were evaluated separately. Exposure information was obtained directly from the Health and Medical Information section of the US Standard Certificate of Live Births.

### Covariates

Baseline variables that were considered clinically relevant or showed a univariate relationship with the outcome were entered into the multivariate logistic regression model (data not shown). Variables for inclusion were carefully chosen to ensure the parsimony of the final model, given the number of events available. Second, candidate variables with *P* < 0.05 on univariate analysis were included in the multivariable model; however, all baseline variables were significant in the univariable logistic regression model due to the large sample size.

The variables included maternal age, race, ethnicity, educational level, marital status, parity, smoking status before and during pregnancy, history of preterm delivery, history of cesarean delivery, pre-pregnancy body mass index (BMI), timing of initiation of prenatal care, prenatal visit counts, WIC (Supplemental Nutrition Program for Women, Infants, and Children), payment for delivery, gestational hypertension, eclampsia, gestational diabetes, neonatal sex, and infection status.

Maternal age was defined as the age at the time of birth and was classified as < 20, 20–24, 25–29, 30–34, 35–39, or ≥ 40 years. Maternal race and ethnicity were categorized as White, Black, Asian, Native American or Alaska Native, Native Hawaiian or other Pacific Islander, people of more than one race, Hispanic, or unknown or unstated racial or ethnic origin. Maternal educational levels were recorded as 8^th^ grade or lower, 9^th^–12^th^ grade without a diploma, 9^th^–12^th^ grade with a diploma, or higher than 12^th^ grade. Marital status was categorized as married or unmarried. Parity, defined as the total number of live births excluding the current delivery, was classified as zero, one, two, three–seven, and eight or more. Smoking status before and during pregnancy was classified as “yes” or “no.” The time of prenatal care initiation was categorized according to the trimester of the first prenatal visit as no prenatal care, first trimester, second trimester, or third trimester. Maternal pre-pregnancy BMI was classified as < 18.5, 18.5– 24.9, 25.0– 29.9, 30.0– 34.9, 35.0– 39.9, or ≥ 40 kg/m^2^. Other risk factors during pregnancy were directly collected from the facility worksheet of the US Standard Certificate of Live Births. Four options for the source of payment at delivery were identified in a checkbox format: 1) private insurance, 2) Medicaid, 3) self-pay, and 4) other (must be specified). Gestational diabetes, gestational hypertension, eclampsia, and history of preterm delivery were classified as “yes” or “no.” These maternal variables were identified from the facility worksheet of the US Standard Certificate of Live Birth (https://www.cdc.gov/nchs/data/dvs/GuidetoCompleteFacilityWks), and diagnostic criteria were documented in the worksheet. When necessary, a missing category for the covariates was added.

### Statistical analysis

Continuous variables are expressed as means and standard deviations, and categorical variables are expressed as frequencies and percentages. A χ^2^ test was used to analyze categorical variables, and an unpaired two-tailed *t*-test or Mann–Whitney U test was used for numerical variables.

In the main analyses, we estimated the association between infertility treatment (ART and non-ART groups separately in the subgroup analysis) and CAM; the natural conception group was used as the reference. Model 1 presents the univariate analysis. Model 2 was adjusted for sociodemographic characteristics, including maternal age, race and ethnicity, educational level, marital status, parity, smoking status before and during pregnancy, history of preterm delivery, history of cesarean delivery, pre-pregnancy BMI, timing of initiation of prenatal care, prenatal visit counts, gestational hypertension, gestational diabetes, eclampsia, neonatal sex, WIC, and payment method. Model 3 included the adjustments for model 2 and was further adjusted for the infection status during pregnancy. All results are presented as odds ratios (OR) and 95% confidence intervals (CI). Several studies have found that CAM is closely related to poor neonatal outcomes, including premature rupture of membranes, preterm birth, and LBW [19]; therefore, we further investigated the effect of infertility treatment on neonatal outcomes in women diagnosed with CAM. Multivariate logistic regression analysis, which was adjusted for the same covariates, was performed to evaluate the association between infertility treatment and neonatal outcomes in women diagnosed with CAM.

Statistical analyses were performed using R, version 4.0.1. All *P*-values were two-tailed, and *P* < 0. 05 was considered statistically significant.

## Results

**Fig. 1:**
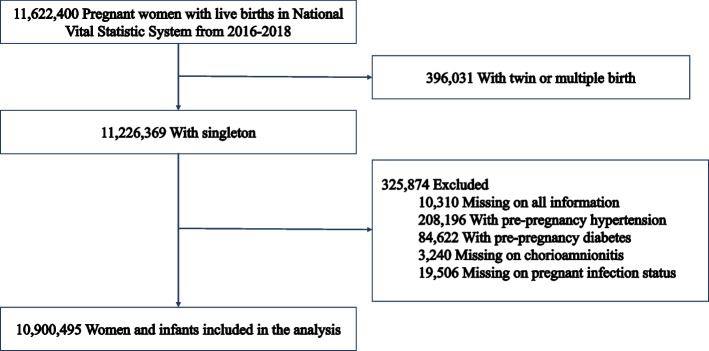
A flow chart of the experiment

The final sample comprised 10,900,495 mothers with singleton live births (Fig. 1). A comparison of the present sample and the excluded individuals is shown in Table S1. Of these women, 151,008 (1.4%) received infertility treatment (Table 1). Compared with the natural conception group, the infertility treatment group tended to be older (34.4 ± 5.3 vs. 28.7 ± 5.8 years), Caucasian (72.4 vs. 51.4%), married (85.9 vs. 54.7%), primipara (42.2 vs. 31.4%), and have a college diploma or higher (90.1 vs. 59.8%). Pre-pregnancy BMI and weight gain during pregnancy were comparable between the two groups. Women receiving infertility treatment tended to take early (1^st^ trimester prenatal care: 88.3 vs. 75.0%) and regular prenatal care visits (prenatal visit count ≥ 16: 10.1 vs. 5.7%) and were more likely to have gestational hypertension (10.1 vs. 6.3%), gestational diabetes (10.3 vs. 6.1%), and CAM (3.0 vs. 1.6%).

**Table 1:** The characteristic of the population

Characteristic	All population	Natural conception	Infertility treatment	*P*-value
2016–2018	10,900,495	10,749,487 (98.6)	151,008 (1.4)	
Years				< .001
2016	3,716,502 (34.1)	3,670,624 (34.1)	45,878 (30.4)	
2017	3,622,835 (33.2)	3,572,006 (33.2)	50,829 (33.6)	
2018	3,561,158 (32.7)	3,506,875 (32.7)	54,301 (36.0)	
Maternal age, Mean (SD)	28.7 ± 5.8	28.7 ± 5.8	34.4 ± 5.3	< .001
Maternal age				
< 20	573,141 (5.3)	559,166 (5.2)	13,975 (8.0)	
20–24	2,201,632 (20.2)	2,159,825 (20.1)	41,807 (24.0)	
25–29	3,195,106 (29.3)	3,143,691 (29.3)	51,415 (29.5)	
30–34	3,076,362 (28.2)	3,031,396 (28.3)	44,966 (25.8)	
≥ 35	1,854,254 (17.0)	1,832,054 (17.1)	22,200 (12.7)	
Race and ethnicity				< .001
White	5,637,228 (51.7)	5,527,876 (51.4)	109,352 (72.4)	
Black	1,523,114 (14.0)	1,516,357 (14.1)	6,757 (4.5)	
AIAN	83,809 (0.8)	83,554 (0.8)	255 (0.2)	
Asia	709,461 (6.5)	692,825 (6.4)	16,636 (11.0)	
NHOPI	26,599 (0.2)	26,512 (0.2)	87 (0.1)	
> 1race	231,508 (2.1)	247,238 (2.3)	2,265 (1.5)	
Hispanic	2,595,187 (23.8)	2,582,707 (24.0)	12,480 (8.3)	
Unknown	93,589 (0.9)	90,579 (0.8)	3,010 (2.0)	
Education				< .001
< 8^th^ grade	356,492 (3.3)	355,975 (3.3)	517 (0.3)	
9–12^th^ grade without diploma	1,085,317 (10.0)	1,083,872 (10.1)	1,445 (1.0)	
High school	2,752,395 (25.2)	2,742,444 (25.5)	9,951 (6.6)	
College or above degree	6,568,372 (60.2)	6,432,282 (59.8)	136,090 (90.1)	
Unknown	137,919 (1.3)	134,914 (1.3)	3,005 (2.0)	
Marital status				< .001
Married	6,009,650 (55.2)	5,879,998 (54.7)	129,652 (85.9)	
Unmarried	4,005,815 (36.7)	3,995,119 (37.2)	10,696 (7.1)	
Unknown	885,030 (8.1)	874,370 (8.1)	10,660 (7.0)	
Parity				< .001
1	3,435,120 (31.5)	3,371,324 (31.4)	63,796 (42.2)	
2	3,072,417 (28.2)	3,031,620 (28.2)	40,797 (27.0)	
3–7	4,165,274 (38.2)	4,120,685 (38.3)	44,589 (29.5)	
≥ 8	182,477 (1.7)	180,990 (1.7)	1,487 (1.0)	
Unknown	45,207 (0.4)	44,868 (0.4)	339 (0.2)	
Pre-pregnancy BMI, (kg/m^2^, Mean ± SD)	26.8 ± 6.5	26.8 ± 6.5	26.3 ± 6.2	< .001
Pre-pregnancy BMI (kg/m^2^)				< .001
< 18.5	367,097 (3.4)	363,669 (3.4)	3,428 (2.3)	
18.5–24.9	4,689,671 (43.0)	4,616,709 (42.9)	72,908 (48.3)	
25–29.9	2,808,007 (25.8)	2,770,212 (25.8)	37,795 (25.0)	
30–34.9	1,538,729 (14.1)	1,519,608 (14.1)	19,121 (12.7)	
35–39.9	733,717 (6.7)	1,724,168 (6.7)	9,549 (6.3)	
≥ 40	495,830 (4.5)	490,045 (4.6)	5,785 (3.8)	
Unknown	267,498 (2.5)	265,076 (2.5)	2,422 (1.6)	
Weight gain (pounds, Mean ± SD)	29.5 ± 14.9	29.5 ± 14.9	29.9 ± 13.5	< .001
Weight gain (pounds)				
< 11	1,010,668 (9.3)	999,935 (9.3)	10,733 (7.1)	
11–20	1,842,421 (16.9)	1,818,969 (16.9)	23,452 (15.5)	
21–30	3, 027,782 (27.7)	2,981,498 (27.7)	46,284 (30.7)	
31–39	2,571,361 (23.6)	2,531,022 (23.6)	40,339 (26.7)	
≥ 40	2,091,552 (19.2)	2,064,705 (19.2)	26,847 (17.8)	
Unknown	356,711 (3.3)	353,358 (3.3)	3,353 (2.2)	
Smoking status				
Pre-pregnancy				< .001
No	9,887,874 (90.7)	9,739,703 (90.6)	148,171 (98.1)	
Yes	961,293 (8.8)	958,732 (8.9)	2,561 (1.7)	
Unknown	51,328 (0.5)	51,051 (0.5)	276 (0.2)	
1^st^ trimester				< .001
No	1,0129,738 (92.9)	9,980,158 (92.8)	149,580 (99.1)	
Yes	719,989 (6.6)	718,837 (6.7)	1,152 (0.7)	
Unknown	50,768 (0.5)	50,492 (0.5)	276 (0.2)	
2^nd^ trimester				< .001
No	10,233,710 (93.9)	10,083,901 (93.8)	149,809 (99.2)	
Yes	615,413 (5.6)	614,493 (5.7)	920 (0.6)	
Unknown	51,372 (0.5)	51,093 (0.5)	279 (0.2)	
3^rd^ trimester				< .001
No	10,250,606 (94.0)	10,101,156 (94.0)	149,450 (99.0)	
Yes	584,163 (5.4)	583,282 (5.4)	881 (0.6)	
Unknown	65,726 (0.6)	65,049 (0.6)	677 (0.4)	
Time of initiation of prenatal care				< .001
1^st^ trimester	8,190,475 (75.1)	8, 057,090 (75.0)	13,385 (88.3)	
2^nd^ trimester	1,759,978 (16.1)	1,747,559 (16.1)	12,419 (8.3)	
3^rd^ trimester	492,141 (4.6)	489,857 (4.6)	2,284 (1.5)	
No	178,189 (1.6)	177,896 (1.7)	293 (0.2)	
Unknown	279,712 (2.6)	277,085 (2.6)	2,627 (1.7)	
Prenatal visit (times)				< .001
No	178,189 (1.6)	177,896 (1.7)	293 (0.2)	
1–8	1,893,391 (17.4)	1,878,128 (17.5)	15,263 (10.1)	
9–12	4,974,243 (45.6)	4,905,661 (45.6)	68,582 (45.4)	
13–16	2,949,575 (27.1)	2,900,909 (27.0)	48,666 (32.2)	
≥ 16	627,812 (5.8)	612,543 (5.7)	15,269 (10.1)	
Unknown	277,285 (2.5)	274,350 (2.5)	2,935 (2.0)	
WIC				< .001
No	6,700,937 (61.5)	6,560,267 (61.0)	140,670 (93.2)	
Yes	4,073,947 (37.3)	4,065,306 (37.8)	8,641 (5.7)	
Unknown	125,611 (1.2)	123,914 (1.2)	1,697 (1.1)	
Payment				< .001
Medicaid	4,616,354 (42.3)	4,607,743 (42.9)	8,611 (5.7)	
Private	5,327,063 (48.9)	5,191,526 (48.3)	135,537 (89.8)	
Self-pay	473,387 (4.3)	470,669 (4.3)	2,718 (1.8)	
Other	419,905 (3.9)	416,385 (3.9)	3,520 (2.3)	
Unknown	63,786 (0.6)	63,164 (0.6)	622 (0.4)	
Gestational diabetes				< .001
No	10,233,331 (93.9)	10,097,926 (93.9)	135,405 (89.7)	
Yes	667,164 (6.1)	651,561 (6.1)	15,603 (10.3)	
Gestational hypertension				< .001
No	10,212,180 (93.7)	10,076,444 (93.7)	135,736 (89.9)	
Yes	688,315 (6.3)	673,043 (6.3)	15,272 (10.1)	
Eclampsia				< .001
No	10,875,230 (99.8)	10,724,672 (99.8)	150,558 (99.7)	
Yes	25,265 (0.2)	24,815 (0.2)	450 (0.3)	
History of preterm birth				0.389
No	10,563,932 (96.9)	10,417,529 (96.9)	146,403 (97.0)	
Yes	336,563 (3.1)	331,958 (3.1)	4,605 (3.0)	
History of cesarean				< .001
No	9,246,679 (84.8)	9,115,508 (84.8)	131,171 (86.9)	
Yes	1,652,813 (15.2)	1,633,979 (15.2)	19,837 (13.1)	
Infection				
Gonorrhea				< .001
No	10,869,158 (99.7)	10,718,199 (99.7)	150,959 (100.0)	
Yes	31,337 (0.3)	31,288 (0.3)	49 (0.0)	
Syphilis				< .001
No	10,889,768 (99.9)	10,738,805 (99.9)	150,963 (100.0)	
Yes	10,727 (0.1)	10,628 (0.1)	45 (0.0)	
Chlamydia				< .001
No	10,700,001 (98.2)	10,549,378 (98.1)	150,623 (99.7)	
Yes	200,494 (1.8)	200,109 (1.9)	385 (0.3)	
Hepatitis B				.009
No	10,876,011 (99.8)	10 725 390 (99.8)	150 621 (99.7)	
Yes	24,484 (0.2)	24 097 (0.2)	387 (0.3)	
Hepatitis C				< .001
No	10,850,932 (99.5)	10,700,040 (99.5)	150,892 (99.9)	
Yes	49,563 (0.5)	49,447 (0.5)	116 (0.1)	
Neonatal sex				0.458
Female	5,322,388 (48.8)	5,248,512 (48.8)	73,876 (48.9)	
Male	5,578,107 (51.2)	5,500,975 (51.2)	77,132 (51.1)	
Chorioamnionitis				< .001
No	10,726,132 (98.4)	10,579,714 (98.4)	146,418 (97.0)	
Yes	174,363 (1.6)	169,773 (1.6)	4,590 (3.0)	

The association between infertility treatment and CAM risk is presented in Table 2. At the population level, crude analyses suggested that women receiving infertility treatment had a higher risk of developing CAM (OR, 1.954 [95% CI, 1.896–2.013]; *P* < 0.001). After adjustment for inclusion year, maternal age, race, ethnicity, and socioeconomic and obstetric covariates (model 2), the odds of CAM were 70% higher in the infertility treatment group than in the natural conception group (adjusted OR [aOR], 1.700 [95% CI, 1.664–1.737]; *P* < 0.001). Model 3 was further adjusted for infection status during pregnancy, and the trend remained (adjusted odds ratio [aOR], 1.772; 95% CI, 1.718–1.827). We further divided infertility treatment into two groups: (1) ART, such as IVF, and (2) non-ART treatment, such as fertility-enhancing drugs. Mothers who received both ART and non-ART treatment were classified into the ART group. Consistent with the main analysis, the ART (aOR, 1.881 [95% CI, 1.810–1.955]; *P* < 0.001) and non-ART groups (aOR, 1.471 [95% CI, 1.394–1.552]; *P* < 0.001) were both associated with a higher risk of CAM (Table S2).

**Table 2:** Odds ratios for the associations between fertility treatment and chorioamnionitis

	Infertility treatment		
	OR	95%CI	*P-*value
Model 1^a^	1.954	1.896–2.013	< .001
Model 2^b^	1.700	1.664–1.737	< .001
Model 3^c^	1.772	1.718–1.827	< .001

^a^Unadjusted model

^b^The model 2 was adjusted for year of inclusion, maternal age, race, education, marital status, parity, smoking status, history of preterm birth, history of cesarean, pre-pregnancy BMI, weight gain, timing of initiation of prenatal care, prenatal visit count, gestational diabetes, gestational hypertension, eclampsia, WIC, payment

^c^Model 2 plus infection status: gonorrhea, syphilis, chlamydia, hepatitis B and hepatitis C

CAM is known to result in short- and long-term effects in newborns [19]. In our study, women diagnosed with CAM delivered babies with poor outcomes (data not shown). However, it is unclear whether infertility treatment worsens the outcomes of newborns suffering from CAM. Therefore, we investigated whether infertility treatment was correlated with a higher risk of adverse neonatal outcomes in women with CAM. In our study, birth weight was lower in the infertility treatment group (3326 ± 693 (g) vs. 3344 ± 629 (g) (Table 3). Furthermore, VLBW was more frequent in the infertility group (3.6 vs. 2.5%). The prevalence of preterm birth was 7.2% in those with natural conception and 9.0% in those who received infertility treatment. The incidence rates of very preterm (1.6 vs. 1.2%) and extremely preterm births (2.9 vs. 1.8%) were significantly higher in the infertility treatment group than in the natural conception group. Neonates conceived after infertility treatment in women with CAM had a higher incidence of NICU admission (32.5 vs. 30.0%, *P* < 0.001), assisted ventilation (13.3 vs. 10.8%, *P* < 0.001), assisted ventilation > 6 h (13.3 vs. 10.8%, *P* < 0.001), surfactant use (1.7 vs. 1.1%, *P* < 0.001), and antibiotic use (31.0 vs. 24.0%, *P* < 0.001).

**Table 3:** Neonatal outcomes of women with chorioamnionitis by infertility treatment

Characteristic	Women diagnosed with CAM	Natural conception	Infertility treatment	*P*- value
Population	174,254 (100.0)	169,668 (97.4)	4,586 (2.6)	
Birth weight (mean) (g)	3,344 ± 631	3,344 ± 629	3,326 ± 693	< .001
Birth weight				< .001
< 1500 g	4,349 (2.5)	4,183 (2.5)	166 (3.6)	
1500–2500 g	6,335 (3.6)	6,184 (3.6)	151 (3.3)	
≥ 2500 g	163,370 (93.8)	159,110 (93.8)	4,260 (92.9)	
Unknown	200 (0.1)	191 (0.1)	9 (0.2)	
Gestational age (mean) (weeks)	38.8 ± 2.8	38.8 ± 2.7	38.6 ± 3.2	
Gestational age				< .001
< 28	3,226 (1.9)	3,105 (1.8)	131 (2.9)	
28–31^+6^	2,140 (1.2)	2,068 (1.2)	72 (1.6)	
32–36^+6^	7,277 (4.2)	7,070 (4.2)	207 (4.5)	
≥ 37	161,547 (92.7)	157,372 (92.8)	4,175 (91.0)	
Unknown	54 (0.0)	53 (0.0)	1 (0.0)	
Apgar 5 min				< .001
	8.5 ± 1.2	8.5 ± 1.2	8.5 ± 1.3	
NICU admission				< .001
	52,348 (30.0)	50,856 (30.0)	1,492 (32.5)	
Assisted ventilation				< .001
	18,960 (10.9)	18,351 (10.8)	609 (13.3)	
Assisted ventilation > 6 h				< .001
	6046 (3.5)	5828 (3.4)	218 (4.8)	
Use of surfactant				< .001
	1,880 (1.1)	1,800 (1.1)	80 (1.7)	
Use of antibiotics				< .001
	42,180 (24.2)	40,760 (24.0)	1,420 (31.0)	
Seizure				0.154
	215 (0.1)	206 (0.1)	9 (0.2)	

^a^The mother-infant pairs without neonatal outcomes records were excluded *N* = 6690 (0.1%). And for gestational age and birth weight, the missing value were retained

We further explored the relationship between infertility treatments and neonatal outcomes (Table 4). The risk of VLBW was 108.3% higher in the infertility treatment group than in the natural conception group (aOR, 2.083 [95% CI, 1.665–2.606], *P* < 0.001). The risk of preterm birth increased by 49.7% in the infertility treatment group (aOR, 1.497 [95% CI, 1.324–1.693]; *P* < 0.001). The same trend was observed for moderate, late, very, and extremely preterm birth. In addition, fetuses delivered by mothers with CAM had a higher risk of NICU admission (aOR, 1.234 [95% CI, 1.156–1.317]; *P* < 0.001), assisted ventilation (aOR, 1.340 [95% CI, 1.224–1.467]; *P* < 0.001), assisted ventilation > 6 h (aOR, 1.603 [95% CI, 1.384–1.856]; *P* < 0.001), surfactant use (aOR, 1.948 [95% CI, 1.524–2.492]; *P* < 0.001), and antibiotic use (aOR, 1.496 [95% CI, 1.400–1.593]; *P* < 0.001). In general, the risk of adverse neonatal outcomes increased in the infertility treatment group with CAM.

**Table 4:** Odds ratio of neonatal outcomes by infertility treatment in chorioamnionitis population

	Model 1^a^	Model 2^b^	Model 3^c^
LBW	0.912 (0.774–1.075)	1.103 (0.927–1.312)	1.103 (0.927–1.312)
VLBW	**1.482** (1.265–1.736)	**2.083** (1.665–2.606)	**2.083** (1.664–2.606)
Preterm birth	**1.262** (1.139–1.399)	**1.497** (1.324–1.692)	**1.497** (1.324–1.693)
Moderate and late preterm birth	1.104 (0.958–1.271)	**1.283** (1.104–1.492)	**1.283** (1.104–1.492)
Very preterm birth	**1.312** (1.035–1.663)	**1.772** (1.356–2.315)	**1.770** (1.355–2.313)
Extremely preterm birth	**1.590** (1.332–1.899)	**2.290** (1.737–3.018)	**2.291** (1.737–3.020)
NICU	**1.127** (1.058–1.199)	**1.233** (1.155–1.315)	**1.234** (1.156–1.317)
Assisted ventilation	**1.263** (1.158–1.377)	**1.339** (1.223–1.466)	**1.340** (1.224–1.467)
Assisted ventilation > 6 h	**1.403** (1.222–1.611)	**1.602** (1.384–1.855)	**1.603** (1.384–1.856)
Use of surfactant	**1.656** (1.321–2.075)	**1.947** (1.522–2.490)	**1.948** (1.524–2.492)
Use of antibiotic	**1.418** (1.331–1.512)	**1.496** (1.400–1.598)	**1.496** (1.400–1.598)

*Abbreviations*: *LBW* Low birth weight, *VLBW* Very low birth weight, *NICU* Neonatal intensive care unit

^a^Unadjusted model

^b^Adjusted for Year, maternal age, race, education, marital status, 2 + parity, smoking status, history of preterm birth, history of cesarean, pre-pregnancy BMI, timing of initiation of prenatal care, prenatal visit count, gestational diabetes, gestational hypertension, eclampsia, fetal sex, gestational age, WIC, and payment for delivery

^c^Model 2 plus infection status: gonorrhea, syphilis, chlamydia, hepatitis B and hepatitis C

Bold value in the table indicated *P* value < 0.05

## Discussion

This nationwide cohort study included almost 11 million pairs of mothers and singleton newborns. We found that women receiving infertility treatment, either ART or non-ART, had significantly greater association with CAM compared with women who underwent natural conception. In addition, newborns delivered by women with CAM in the infertility group had a higher risk of adverse outcomes such as preterm birth, LBW, and NICU admission before discharge from the hospital compared with ones conceived naturally.

In recent decades, infertility has become an emerging common health issue and has attracted considerable attention [20]. Despite the increased availability and usage of fertility treatment worldwide, its potential adverse effects, including CAM, have not been fully explored. Consistent with our results, a retrospective cohort study exploring the pregnancy outcomes of frozen-thawed IVF with classified infertility etiology found that IVF pregnancies were related to a higher rate of CAM (2.93 [1.04–8.26]) than spontaneous pregnancies [18]. Another study evaluating the mediating effects of multiple gestations on pregnancy complications indicated that women who received treatment during pregnancy had a higher risk of CAM in both the direct and mediated pathways [21]. Pregnant women manifested with any combination of fever, maternal or fetal tachycardia, uterine tenderness, foul-smelling amniotic fluid, or an elevated white blood cell count are suspected to have CAM. However, the presence of one (or more) of these signs and symptoms is not always related to the occurrence of intra-uterine inflammation or the histopathologic CAM. A study found 24% patients diagnosed with preterm birth and clinical CAM had no evidence of either intra-amniotic infection or inflammation, and only 34% had positive amniotic fluid cultures. Patients without microbial invasion of the amniotic cavity or intra-amniotic inflammation had lower rates of adverse outcomes than those exposed to the infection or inflammation [22]. Due to the limitations of the database in the current study, clinical CAM was referred to as exposure, which might not be consistent with “golden evidence” from histological results. Imprecise definitions and variable clinical manifestations, as discussed above, are loosely used to label a heterogeneous array of conditions characterized by infection and inflammation or both. Further studies should explore the relationship between histological CAM and infertility treatment in order to provide clinical management for mothers with CAM and their newborns.

CAM is closely related to preterm birth [2]. The risk of preterm birth resulting from IVF/ICSI is known to be significantly greater than that in spontaneously conceived pregnancies, especially in singleton pregnancies [23–25]. In our study, we investigated whether infertility treatment worsens neonatal outcomes in women already diagnosed with CAM. Interestingly, infertility treatment increased all adverse neonatal outcomes, indicating a synergistic effect between the two exposures. Heterogeneous infertility factors such as endometriosis, adenomyosis, polycystic ovary syndrome, and uterine fibroids already exist; thus, inflammatory pathways, hormonal aberrations, decidual senescence, and vascular abnormalities that may impair pregnancy success might increase the adverse effect of infertility treatment. Except for preterm birth, patients with preterm premature rupture of membranes (PPROM) are apt to develop CAM during expectant management [26]; however, the relationship between ART and PPROM was inconsistent. A study found that IVF/ICSI pregnancies were associated with a decreased risk of PPROM (aOR, 0.64; 95% CI 0.42–0.99) [27]; meanwhile, a meta-analysis suggested no differences in the risk of PPROM among women after fresh embryo transplantation and those undergoing natural conception (Risk Ratio 0.92, 95% CI 0.72–1.18; I^2^ = 0%) [28]. The reason for this remains unclear, and the database did not include PPROM diagnoses.

The potential mechanisms underlying the association between infertility treatments and CAM remain unclear. Despite demonstrable microorganisms, CAM can occur as “sterile” intra-amniotic inflammation under conditions of cellular stress, injury, or death [29] and is induced by environmental pollutants [30], cigarette smoke [31], and ART. A Previous study has shown that ART is linked to dysregulated inflammation and oxidative stress in an assisted reproductive mouse model [32]. Specifically, greater levels of apoptosis and degraded nucleotides, accompanied by higher interleukin (IL)-6 concentrations, were observed in ART placentas, indicating inflammatory status and cellular stress. Placentas from mouse pregnancies achieved by ART also had lower activity of antioxidant enzymes such as superoxide dismutase, glutathione-S-transferase, and xanthine oxidase, which are more severe in pregnancies fertilized using ICSI [33]. Another proposed mechanism is that ART can influence the period around conception when widespread epigenetic changes occur [34]. Furthermore, steroid diffusion/flow from the mother to the fetus is altered in murine pregnancies conceived by ART [33], which may affect trophoblast function in early pregnancy during implantation and placentation. Further research on the potential biological mechanisms underlying CAM resulting from infertility treatment is necessary.

A consequence of ART is a progressive rise in the incidence of twin, triplet, and multiple pregnancies. To avoid potential bias, this study focused on singleton pregnancies. Several studies have reported that ART-induced pregnancies, whether singleton or multiple, have an increased risk of preterm birth and LBW compared with pregnancies that conceived naturally [35], which can also result from CAM. In this study, we found that newborns delivered by women with CAM who received infertility treatment had a higher risk of preterm birth, VLBW, NICU admission, and other supportive treatments. This suggests that infertility treatment could lead to adverse perinatal outcomes apart from CAM. A recent study found that cytokine levels, such as IL-1β, IL-6, and IL-8, were increased in natural conception pregnancies complicated by CAM. Conversely, interferon-γ and tumor necrosis factor-α were decreased in ART [17], which might explain the more inflexible status related to adverse neonatal outcomes in newborns exposed to both infertility treatment and CAM.

### Strengths and limitations

This study has several strengths. This study analyzed a nationwide population of mother-newborn pairs, providing sufficient statistical power to examine the association between maternal infertility treatment and CAM. Furthermore, abundant data allowed us to include most confounding factors, such as infection status during pregnancy, to validate our results. However, this study had several limitations. First, the study did not consider the etiology of infertility diagnoses, such as ovulation disorder, tubal disease, endometriosis, male infertility, or mixed infertility (i.e., multiple infertility-related diagnoses). Second, although we adjusted for many maternal characteristics and complicated pregnancy conditions to mitigate confounding factors, we used administrative data, and thus could not guarantee complete and accurate data collection.

## Conclusions

Our study results indicate that women receiving infertility treatment were associated with a higher risk of CAM than women who conceived naturally. Furthermore, newborns exposed to CAM in the infertility treatment group were at an increased risk of preterm birth, VLBW, and NICU admission compared with ones conceived naturally. Further investigations are warranted to elucidate the mechanisms underlying the association between infertility treatment and risk of CAM.

## Supplementary Information

**Additional file 1: Table S1.** The Baseline Comparison Between Included and Excluded Participants. **Table S2.** Odds Ratios for the Associations Between Infertility Treatment (ART and Non-ART) and Chorioamnionitis.

## Data Availability

Public-use data can be downloaded from the NVSS website (https://www.cdc.gov/nchs/data_access/vitalstatsonline.htm).

## Abbreviations

CAM: Chorioamnionitis
OR: Odds ratio
aOR: Adjusted odds ratio
CI: Confidence interval
NICU: Neonatal intensive care unit
ART: Assisted reproductive technology
IVF: In vitro fertilization
ICSI: Intracytoplasmic sperm injection
LBW: Low birth weight
VLBW: Very low birth weight
BMI: Body mass index
PPROM: Preterm premature rupture of membranes
US: United States
IL: Interleukin
