# Supplementary material for: Association of chorioamnionitis with infertility treatment and subsequent neonatal outcomes in the US: a population-based cohort study
Source: BMC Pregnancy Childbirth. 2023 May 20;23:369. doi: 10.1186/s12884-023-05619-0 (PMC10200046; doi:10.1186/s12884-023-05619-0)
Supplement: Supplementary file 1 — Additional file 1: Table S1. The Baseline Comparison Between Included and Excluded Participants. Table S2. Odds Ratios for the Associations Between Infertility Treatment (ART and Non-ART) and Chorioamnionitis. [file 12884_2023_5619_MOESM1_ESM.docx]

**Table S1. The Baseline Comparison Between Included and Excluded Participants.**

|  |  |  |  |  |
| --- | --- | --- | --- | --- |
| Characteristic | All population | Included | Excluded | *P*-value |
| 2016-2018 | 11,622,400 (100.0) | 10,900,495 (93.8) | 721,905 (6.2) |  |
| Years |  |  |  | <.001 |
| 2016 | 3,956,112 (34.0) | 3,716,502 (34.1) | 239,610 (33.2) |  |
| 2017 | 3,864,754 (33.3) | 3,622,835 (33.2) | 241,919 (33.5) |  |
| 2018 | 3,801,534 (32.7) | 3,561,158 (32.7) | 240,376 (33.3) |  |
| Maternal age, Mean (SD) |  |  |  | <.001 |
| <20 | 591,137 (5.1) | 573,141 (5.3) | 17,996 (2.5) |  |
| 20-24 | 2,299,347 (19.8) | 2,201,632 (20.2) | 97,715 (13.5) |  |
| 25-29 | 3,380,883 (29.1) | 3,195,106 (29.3) | 185,777 (25.7) |  |
| 30-34 | 3,302,735 (28.4) | 3,076,362 (28.2) | 226,373 (31.4) |  |
| ≥35 | 2,048,298 (17.6) | 1,854,254 (17.0) | 194,044 (26.9) |  |
| Race and ethnicity |  |  |  | <.001 |
| White | 6,007,884 (51.7) | 5,637,228 (51.7) | 370,656 (51.3) |  |
| Black | 1,673,162 (14.4) | 1,523,114 (14.0) | 150,048 (20.8) |  |
| AIAN | 90,547 (0.8) | 83,809 (0.8) | 6,738 (0.9) |  |
| Asia | 745,709 (6.4) | 709,461 (6.5) | 36,248 (5.0) |  |
| NHOPI | 28,267 (0.2) | 26,599 (0.2) | 1,668 (0.2) |  |
| >1race | 247,298 (2.1) | 231,508 (2.1) | 15,790 (2.2) |  |
| Hispanic | 2,726,356 (23.5) | 2,595,187 (23.8) | 131,169 (18.2) |  |
| Unknown | 103,177 (0.9) | 93,589 (0.9) | 9,588 (1.3) |  |
| Education |  |  |  | <.001 |
| < 8^th^ | 375,596 (3.2) | 356,492 (3.3) | 19,104 (2.6) |  |
| 9-12^th^ without diploma | 1,148,180 (9.9) | 1,085,317 (10.0) | 63,862 (8.7) |  |
| High school | 2,924,219 (25.2) | 2,752,395 (25.3) | 171,824 (23.8) |  |
| College or above degree | 7,023,675 (60.4) | 6,568,372 (60.3) | 455,303 (63.1) |  |
| Unknown | 150,730 (1.3) | 137,919 (1.3) | 12,811 (1.8) |  |
| Marital status |  |  |  | <.001 |
| Married | 6,423,513 (55.3) | 6,009,650 (55.1) | 413,863 (57.3) |  |
| Unmarried | 4,269,984 (36.7) | 4,005,815 (36.7) | 264,169 (36.6) |  |
| Unknown | 928,903 (8.0) | 885,030 (8.1) | 43,873 (6.1) |  |
| Parity |  |  |  | <.001 |
| 1 | 3,590,395 (30.9) | 3,435,120 (31.5) | 155,275 (21.5) |  |
| 2 | 3,263,828 (28.1) | 3,072,417 (28.2) | 191,411 (26.5) |  |
| 3-7 | 4,510751 (38.8) | 4,165,274 (38.2) | 345,477 (47.9) |  |
| ≥ 8 | 204,962 (1.8) | 182,477 (1.7) | 22,485 (3.1) |  |
| Unknown | 52,464 (0.5) | 45,207 (0.4) | 7,257 (1.0) |  |
| Pre-pregnancy BMI |  |  |  | <.001 |
| < 18.5 | 380,399 (3.3) | 367,097 (3.4) | 13,302 (1.8) |  |
| 18.5-24.9 | 4,904,366 (42.2) | 4,689,617 (43.0) | 214,749 (29.7) |  |
| 25-29.9 | 2,979,468 (25.6) | 2,808,007 (25.8) | 1,171,461 (23.8) |  |
| 30-34.9 | 1,666,164 (14.3) | 1,538,729 (14.1) | 127,435 (17.7) |  |
| 35-39.9 | 816,705 (7.0) | 733,717 (6.7) | 82,988 (11.5) |  |
| ≥ 40 | 582,498 (5.0) | 495,830 (4.5) | 86,668 (12.0) |  |
| Unknown | 292,800 (2.5) | 267,498 (2.5) | 25,302 (3.5) |  |
| Weight gain |  |  |  | <.001 |
| Weight gain |  |  |  |  |
| < 11 | 1,091,396 (9.4) | 1,010,668 (9.3) | 80,728 (11.2) |  |
| 11-20 | 1,952,539 (16.8) | 1,842,421 (16.9) | 110,118 (15.3) |  |
| 21-30 | 3,180,200 (27.4) | 3,027,782 (27.8) | 152,418 (21.1) |  |
| 31-40 | 2,717,967 (23.4) | 2,571,361 (23.6) | 146,606 (20.3) |  |
| > 40 | 2,288,872 (19.7) | 2,091,552 (19.2) | 193,720 (27.3) |  |
| Unknown | 391,426 (3.4) | 356,711 (3.3) | 34,715 (4.8) |  |
| Smoking status |  |  |  |  |
| Pre-pregnancy |  |  |  | <.001 |
| No | 10,532,850 (90.6) | 9,887,874 (90.7) | 644,976 (89.3) |  |
| Yes | 1,032,887 (8.9) | 961,293 (8.8) | 71,594 (9.9) |  |
| Unknown | 56,663 (0.5) | 51,328 (0.5) | 5,335 (0.7) |  |
| 1^st^ trimester |  |  |  | <.001 |
| No | 10,792,157 (92.9) | 10,129,738 (92.9) | 662,419 (91.8) |  |
| Yes | 774,126 (6.7) | 719,989 (6.6) | 54,137 (7.5) |  |
| Unknown | 56,117 (0.5) | 50,768 (0.5) | 5,349 (0.7) |  |
| 2^nd^ |  |  |  | <.001 |
| No | 10,903,895 (93.8) | 10,233,710 (93.9) | 670,185 (92.8) |  |
| Yes | 661,717 (5.7) | 615,413 (5.6) | 46,304 (6.4) |  |
| Unknown | 56,788 (0.5) | 51,372 (0.5) | 5,416 (0.8) |  |
| 3^rd^ |  |  |  | <.001 |
| No | 10,918,419 (93.9) | 10,250,606 (94.0) | 667,813 (92.5) |  |
| Yes | 627,464 (5.4) | 584,163 (5.4) | 43,301 (6.0) |  |
| Unknown | 76,517 (0.7) | 65,726 (0.6) | 10,791 (1.5) |  |
| Time of initiation of prenatal care |  |  |  | <.001 |
| 1^st^ | 8,738,543 (75.2) | 8,190,475 (75.1) | 548,068 (75.9) |  |
| 2^nd^ | 1,866,540 (16.1) | 1,759,978 (16.1) | 106,562 (14.8) |  |
| 3^rd^ | 516,160 (4.4) | 492,141 (4.5) | 34,019 (3.3) |  |
| No | 193,361 (1.7) | 178,189 (1.6) | 15,162 (2.1) |  |
| Unknown | 307,806 (2.6) | 279,712 (2.6) | 28,094 (3.9) |  |
| Prenatal visit |  |  |  | <.001 |
| No | 193,351 (1.7) | 178,189 (1.6) | 15,162 (2.1) |  |
| 1-8 | 2,033,442 (17.5) | 1,893,391 (17.4) | 140,051 (19.4) |  |
| 9-12 | 5,234,511 (45.0) | 4,974,243 (45.6) | 260,268 (36.1) |  |
| 13-16 | 3,123,932 (26.9) | 2,949,575 (27.1) | 174,357 (24.2) |  |
| ≥ 16 | 730,857 (6.3) | 727,812 (5.8) | 103,045 (14.3) |  |
| Unknown | 306,307 (2.6) | 277,285 (2.5) | 29,022 (4.0) |  |
| WIC |  |  |  | <.001 |
| No | 7,135,750 (61.4) | 6,700,937 (61.5) | 434,813 (60.2) |  |
| Yes | 4,249,435 (37.4) | 4,073,947 (37.4) | 275,488 (38.2) |  |
| Unknown | 137,215 (1.2) | 125,611 (1.2) | 11,604 (1.6) |  |
| Payment |  |  |  | <.001 |
| Medicaid | 4,915,383 (42.3) | 4,616,354 (42.3) | 299,029 (41.4) |  |
| Private | 5,691,754 (49.0) | 6,237,063 (48.9) | 364,691 (50.5) |  |
| Self-pay | 496,505 (4.3) | 473,387 (4.3) | 23,118 (3.2) |  |
| Other | 447896 (3.9) | 419,905 (3.9) | 27,991 (3.9) |  |
| Unknown | 70,862 (0.6) | 63,786 (0.6) | 7,076 (1.0) |  |
| Gestational diabetes |  |  |  | <.001 |
| No | 10,878,724 (93.6) | 10,233,331 (93.9) | 645,393 (89.4) |  |
| Yes | 733,063 (6.3) | 667,164 (6.1) | 65,899 (9.1) |  |
| Unknown | 10,613 (0.1) | 0 | 10,613 (1.5) |  |
| Gestational hypertension |  |  |  | <.001 |
| No | 10,855,058 (93.4) | 10,212,180 (93.7) | 642,878 (89.1) |  |
| Yes | 756,729 (6.5) | 688,315 (6.3) | 68,414 (9.5) |  |
| Unknown | 10,613 (0.1) | 0 | 10,613 (0.1) |  |
| Eclampsia |  |  |  | <.001 |
| No | 11,581,242 (99.6) | 10,875,230 (99.8) | 706,012 (97.8) |  |
| Yes | 30,545 (0.3) | 25,265 (0.2) | 5,280 (0.7) |  |
| Unknown | 10,613 (0.1) | 0 | 10,613 (0.1) |  |
| History of preterm birth |  |  |  | 0.389 |
| No | 11,228,799 (96.6) | 10,563,932 (96.9) | 664,867 (92.1) |  |
| Yes | 382,988 (3.3) | 336,563 (3.1) | 46,425 (6.4) |  |
| Unknown | 10,613 (0.1) | 0 | 10,613 (0.1) |  |
| History of cesarean |  |  |  | <.001 |
| No | 9,812,131 (84.4) | 9,346,679 (84.8) | 565,452 (78.3) |  |
| Yes | 1,799,656 (15.5) | 1,653,816 (15.2) | 145,840 (20.2) |  |
| Unknown | 10,613 (0.1) | 0 | 10,613 (0.1) |  |
| Infection |  |  |  |  |
| Gonorrhea |  |  |  | <.001 |
| No | 11,560,348 (99.5) | 10,869,158 (99.7) | 691,190 (95.7) |  |
| Yes | 33,638 (0.3) | 31,337 (0.3) | 2,301 (0.3) |  |
| Unknown | 28,414 (0.2) | 0 | 28,414 (0.2) |  |
| Syphilis |  |  |  | <.001 |
| No | 11,582,232 (99.7) | 10,889,768 (99.9) | 692,464 (96.9) |  |
| Yes | 11,754 (0.1) | 10,727 (0.1) | 1,027 (0.1) |  |
| Unknown | 28,414 (0.2) | 0 | 28,414 (0.2) |  |
| Chlamydia |  |  |  | <.001 |
| No | 11,381,950 (97.9) | 10,700,001 (98.2) | 681,949 (94.5) |  |
| Yes | 212,036 (1.8) | 200,494 (1.8) | 11,542 (1.6) |  |
| Unknown | 28,414 (0.2) | 0 | 28,414 (0.2) |  |
| Hepatitis B |  |  |  | <.001 |
| No | 11,567,830 (99.5) | 10,876,011 (99.8) | 691,819 (95.8) |  |
| Yes | 26,156 (0.2) | 24,484 (0.2) | 1,672 (0.2) |  |
| Unknown | 28,414 (0.2) | 0 | 28,414 (0.2) |  |
| Hepatitis C |  |  |  | <.001 |
| No | 11,540,355 (99.3) | 10,850,932 (99.5) | 689,423 (95.5) |  |
| Yes | 53,631 (0.5) | 49,563 (0.5) | 4,068 (0.6) |  |
| Unknown | 28,414 (0.2) | 0 | 28,414 (0.2) |  |
| Neonatal sex |  |  |  | <.001 |
| Female | 5,678,015 (48.9) | 5,322,388 (48.8) | 355,627 (49.3) |  |
| Male | 5,944,385 (51.1) | 5,578,107 (51.2) | 366,278 (50.7) |  |
| Chorioamnionitis |  |  |  | <.001 |
| No | 11,433,031 (98.4) | 10,726,132 (98.4) | 706,899 (97.9) |  |
| Yes | 182,797 (1.6) | 174,363 (1.6) | 8,434 (1.2) |  |
| Unknown | 6,572 (0.1) | 0 | 6,572 (0.1) |  |
| Infertility treatment |  |  |  | <.001 |
| No | 11,396,535 (98.1) | 10,749,487 (98.6) | 647,048 (89.6) |  |
| Yes | 215,252 (1.9) | 151,008 (1.4) | 64,244 (8.9) |  |
| Unknown | 10,613 (0.1) | 0 | 10,613 (0.1) |  |

**Table S2.** **Odds Ratios for the Associations Between Infertility Treatment (ART and Non-ART) and Chorioamnionitis**

|  | Infertility treatment | | | | | |
| --- | --- | --- | --- | --- | --- | --- |
|  | ART | | | Non-ART | | |
|  | OR | 95%CI | *P-*value | OR | 95%CI | *P-*value |
| Model 1^a^ | 2.163 | 2.083-2.245 | <.001 | 1.696 | 1.609-1.788 | <.001 |
| Model 2^b^ | 1.883 | 1.812-1.957 | <.001 | 1.471 | 1.394-1.552 | <.001 |
| Model 3^c^ | 1.881 | 1.810-1.955 | <.001 | 1.471 | 1.394-1.552 | <.001 |

1. Univariable
2. Year of inclusion, maternal age, race, education, marital status, parity, smoking status, history of preterm birth, history of cesarean, pre-pregnancy BMI, weight gain, timing of initiation of prenatal care, prenatal visit count, gestational diabetes, gestational hypertension, eclampsia, WIC, payment,
3. Model 2 plus infection status: gonorrhea, syphilis, chlamydia, hepatitis B and hepatitis C
